# Feasibility of up-regulating CD4^+^CD25^+^ Tregs by IFN-γ in myasthenia gravis patients

**DOI:** 10.1186/s12883-015-0419-9

**Published:** 2015-09-07

**Authors:** Shuo Huang, Weizhi Wang, Lijun Chi

**Affiliations:** Department of Neurology, The First Affiliated Hospital of Harbin Medical University, Harbin, 150080 P.R. China; Department of Neurology, The Second Affiliated Hospital of Harbin Medical University, Harbin, 150086 P.R. China

## Abstract

**Background:**

In myasthenia gravis (MG) patients, the dysfunction of CD4^+^CD25^+^ regulatory T cells (CD4^+^CD25^+^ Tregs) may be one of the important pathogenesis of MG. Currently, the role of IFN-γ in autoimmune diseases is still controversial and needs further exploration. In this study, whether IFN-γ can induce CD4^+^CD25^−^ T cells into CD4^+^CD25^+^ Tregs in MG in vitro was investigated systematically.

**Methods:**

Flow cytometry was used to analyze the number of CD4^+^CD25^+^ Tregs in MG patients and healthy controls (HCs). CD4^+^CD25^−^ T cells were separated from the peripheral blood mononuclear cells of MG patients and HCs, and the CD4^+^CD25^+^ Tregs were separated from HCs by Magnetic cell sorting (MACS). IFN-γ with different concentrations was used to stimulate CD4^+^CD25^−^ T cells. The percentages of the induced CD4^+^CD25^+^ T cells were detected by flow cytometry. The FoxP3 expression of the induced CD4^+^CD25^+^ T cells in MG patients was detected by real-time PCR at mRNA level. The induced CD4^+^CD25^+^ T cells were co-cultured with autologous CD4^+^CD25^−^ T cells to estimate the suppressive ability of the induced CD4^+^CD25^+^ T cells to CD4^+^CD25^−^ T cells.

**Results:**

It shows the percentages of CD4^+^CD25^+^ T cells among CD4^+^ T cells have no significant difference in MG patients compared with those in HCs. There is also merely no difference in the percentages of CD4^+^CD25^+^ T cells between thymectomized and non-thymectomized MG patients. CD4^+^CD25^−^ T cells can be induced to CD4^+^CD25^+^ T cells after applying IFN-γ in MG patients and HCs. The proportion and FoxP3 expression of the induced CD4^+^CD25^+^ T cells are the highest at the level of 40 ng/ml IFN-γ, and the suppressive function of the CD4^+^CD25^+^ T cells induced by 40 ng/ml IFN-γ is the strongest in MG patients.

**Conclusions:**

This subject will further reveal the role of IFN-γ in the pathogenesis of MG from a new perspective. It will also provide the scientific basis for the clinical targeted therapy of MG.

## Background

Myasthenia gravis (MG) is an autoimmune disorder characterized by muscle weakness and chronic fatigue, which results from a blockage of the nerve impulse transmission from nerve endings to muscles by anti-acetylcholine receptor (AchR) antibodies at the neuromuscular junction. In 1895, the Germany doctor Jolly first officially named MG. But up till now, the exact etiology and pathogenesis of MG remain unclear. Therefore, it is key to explore the pathogenesis and search for the therapeutic targets of MG. And the results may suggest one important way to further overcome the other autoimmune diseases.

As the most important regulatory T cells, CD4^+^CD25^+^ regulatory T cells (CD4^+^CD25^+^ Tregs) were firstly defined by Sakaguchi in 1995 and they are characterized by expressing the chain of IL-2 receptor alpha (CD25) and fork head transcription factor 3 (FoxP3) [1]. CD4^+^CD25^+^ Tregs can suppress the potential autoreactive T cells in a positive manner [1] and protect the body from CD4^+^ T cell-mediated autoimmune diseases [2–4]. Therefore, it suggests it is possible to find the targets that influence the attack of MG in regulatory T cells.

Previous studies once showed that the imbalance between Th1 and Th2 can result in MG. But with the discovery of CD4^+^CD25^+^ Tregs, researchers had a new understanding about MG [5]. It is noted that in MG patients, if insufficient CD4^+^CD25^+^ Tregs are produced to suppress the autoreactive T cells, the disease will be aggravated, while if the body can produce enough CD4^+^CD25^+^ Tregs against their autoreactive T cells, the disease will be alleviated [6]. The experimental results showed that in the animal model of MG (experimental autoimmune myasthenia gravis, EAMG), the number of CD4^+^CD25^+^ Tregs in CD4^+^ T spleen cells decreased and the expression of FoxP3 at mRNA level also decreased correspondingly [7]. The first part of our study mainly showed that the number of CD4^+^CD25^+^ T cells in MG patients has no statistical difference from the number of healthy controls (HCs), but the function of them was seriously destroyed, which is consistent with the results of Luther and his colleagues.

At present, there is still inadequacy of previous research in explaining the molecular basis about the generation, development and function of CD4^+^CD25^+^ Tregs. Although recent studies have found that TGF-β, GITR, CTLA 4 and IL-10 are related to its function [8], these molecules are not specific for CD4^+^CD25^+^ Tregs. Many research groups have pointed out that FoxP3 plays an important role in the development and function of CD4^+^CD25^+^ Tregs [9, 10]. The expression of FoxP3 is a necessary and sufficient condition for the development and function of CD4^+^CD25^+^ Tregs. Hence, in our study it is prerequisite to determine whether the induced CD4^+^CD25^+^ T cells are consistent with CD4^+^CD25^+^ Tregs of HCs on phenotype and whether the induced CD4^+^CD25^+^ T cells can express FoxP3 besides CD25. CD4^+^CD25^+^ Tregs were once considered to be generated from thymus only. However, many studies have shown that CD4^+^CD25^+^ Tregs can be generated peripherally [11]. Walker and his colleagues reported that in vitro the expression of FoxP3 can be induced after 24 h by stimulating CD4^+^CD25^−^ T cells from peripheral blood with anti-CD3 and anti-CD28 antibodies, and the expression of FoxP3 reaches its peak after 72 h. And the induced CD4^+^CD25^+^ T cells in vitro have the similar function as naturally occurring CD4^+^CD25^+^ Tregs [10]. Many other studies validated that in vitro the CD4^+^CD25^−^ T cells from mice co-culture with IL-2, TGF-β, anti-CD3 and anti-CD28 antibodies can induce the expression of FoxP3 and make them obtain the regulating activity [12, 13]. Therefore, exploring the methods of inducing CD4^+^CD25^+^ Tregs can make up for the defective function of CD4^+^CD25^+^ Tregs in MG patients. It may provide a new strategy for treating MG and new drug targets. It is also helpful to further study the pathogenesis of MG.

IFN-γ is regarded as a pro-inflammatory cytokine. However the question remains about the exact role of IFN-γ in EAMG. On one hand, it is not easy to make EAMG model for the mice lack of IFN-γ receptor. On the other hand, it is easier to build EAMG model in IFN-γ-deficient mice [14]. The paradoxical experimental evidence indicates that IFN-γ is a “double-edged sword”. Different concentrations, micro environments or stages of immune responses lead to different effects. It promotes a Th1 response and T cell migration to the site of inflammation and amplifies an inflammatory cascade through its ability to mediate a variety of signaling events, causing the production of inflammatory molecules [15]. Moreover, the intensity of Th1-induced inflammation as quantifiable by the production of IFN-γ potentially triggers the immune system to call for a controlling mechanism.

Present study indicated that IFN-γ can induce CD4^+^CD25^−^ T cells to CD4^+^CD25^+^ Tregs in vitro in GBS patients [16]. MG is also an autoimmune disease as GBS and the function of CD4^+^CD25^−^ T cells has no difference between MG and GBS. Thus, in the second part of our study, we put forward and focus our study on the hypothesis “ IFN-γ can induce CD4^+^CD25^−^ T cells into CD4^+^CD25^+^ Tregs in MG patients to make up for the defective function of CD4^+^CD25^+^ Tregs from MG patients.” This topic will further reveal the role of IFN-γ in the pathogenesis of MG patients, providing theoretical and scientific basis for clinical further targeting treatment of MG.

## Methods

### Patients and specimens

Twenty three patients (11 females and 12 males, aged 23–79 years) who met the criteria of MG were from the First Affiliated Hospital of Harbin Medical University during 2012 to 2014. Among them, ten patients were thymectomized at least 1 year because of thymic hyperplasia or thymoma, while other 13 patients have no abnormality of thymus. Prior to this study, all of the patients had not received the treatment of corticosteroids and other immunodepressants at least 1 year in advance to avoid any interference effects [17]. In addition, the patients with chronic-immune-mediated disorders or those who had received any immunomodulatory drugs within 3 months were excluded. The peripheral blood samples were obtained prior to the start of treatment. The characteristics of all the subjects are displayed in Table 1. 19 healthy donors (ten females and nine males, aged 18–70 years) were included as healthy controls (HCs). Ethical approval for this study was granted by the Medical Ethics Review Board of the First Affiliated Hospital of Harbin Medical University and informed consent was obtained from every donor.

**Table 1:** Clinical details of MG patients

No.	Sex	Age	Osserman’s classification	Thymic history
1	F	23	IIA	Thymic hyperplasia
2	F	23	I	_
3	M	27	I	_
4	F	28	IIA	Thymoma
5	M	32	I	_
6	M	33	I	_
7	F	35	IIA	_
8	M	45	IIA	Thymoma
9	M	48	I	Thymic hyperplasia
10	M	48	IIA	Thymoma
11	F	50	IIA	_
12	F	55	IIA	Thymic hyperplasia
13	M	58	IIA	_
14	M	59	IIA	_
15	M	62	I	_
16	F	63	IIA	Thymic hyperplasia
17	M	67	I	_
18	M	67	IIA	_
19	F	69	IIB	Thymoma
20	F	69	IIA	Thymoma
21	M	73	IV	_
22	F	76	IIB	Thymic hyperplasia
23	F	79	IIB	_

### Isolation and purification of T-cell subsets

The peripheral blood mononuclear cells were separated by Ficoll density centrifugation (Nycomed, Oslo, Norway). A human CD4^+^CD25^+^ Treg isolation kit (Miltenyi Biotec) was used according to the protocol to isolate CD4^+^CD25^−^ T cells from MG patients. And naturally occurring CD4^+^CD25^+^ Tregs (nTregs) are isolated from healthy donors by Magnetic cell sorting (MACS). The purity of these subpopulations was assessed by flow cytometry. It exceeded 96 % for CD4^+^CD25^−^ T cells and was more than 93 % for nTregs. The non-CD4^+^ T cells from healthy donors were irradiated (3,000 rads) as antigen-presenting cells (APCs).

### Conversion of CD4^+^CD25^−^ T cells to induced CD4^+^CD25^+^ T cells

CD4^+^CD25^−^ T cells from MG patients and HCs were cultured in RPMI medium (supplemented with 10 % fetal calf serum, L-glutamine, penicillin/streptomycin) at 2 × 10^6^ cells/ml with the indicated concentrations (0, 20, 40, 80 ng/ml) of recombinant human IFN-γ (Peprotech) respectively in the presence of anti-CD3 antibody (1 μg/ml; Clone: HIT3a, Biolegend) and anti-CD28 antibody (1 μg/ml; Clone: CD28.2, Biolegend) in 96-well flat-bottom plates at a final volume of 200 μl. After 3 days, the resulting T cells were harvested and washed twice with medium to remove residual IFN-γ. MACS was used again to purify the induced CD4^+^CD25^+^ T cells in the resulting T cells. The purity of the induced CD4^+^CD25^+^ T cells was more than 93 % by flow cytometry. As described below, the induced CD4^+^CD25^+^ T cells refer to the CD4^+^CD25^+^ cells induced from CD4^+^CD25^−^ T cells in MG patients and nTregs refer to the CD4^+^CD25^+^ Tregs from healthy donors.

### Monoclonal antibodies and flow cytometric analysis

Flow cytometric analysis was performed to quantify the induced CD4^+^CD25^+^ T cells. Cells were stained with PerCP-conjugated anti-CD4 (Clone: L200, BD Pharmingen), FITC-conjugated anti-CD25 (Clone: M-A251, BD Pharmingen) and PE-conjugated anti-FoxP3 (Clone: 206D, BD Pharmingen) respectively. Cells were stained with propidium iodide (PI, Sigma-aldrich) for nonviable cell exclusion prior to being analyzed on a FACS Calibur cytometerical (Becton Dickinson). Data processing was accomplished by CELLQuest software (BD). Cell viability measured by PI was always more than 98 % before stimulating and exceeded 95 % after stimulating.

### Proliferation assay

Co-cultures were established in 96-well U-bottom plates incubated with 2 μg/ml anti-CD3 antibody. CD4^+^CD25^−^ T cells (2 × 10^4^ cells) were cultured with either the induced CD4^+^CD25^+^ T cells or nTregs (2 × 10^4^ cells). 1.0 × 10^5^ irradiated APCs were added to each well and all cells were cultured in a final volume of 200 μl. Cells were incubated at 37 °C for 3 days and then pulsed with 1 μCi [^3^H]-thymidine per well for the final incubation for 18 h. Plates were harvested onto nylon filters using the Betaplate system and radioactivity was quantified using a Betaplate counter. Results are expressed in counts per minute (cpm) as the mean of triplicate cultures ± SEM. The suppressive percentage was calculated using the formula: (1 − cpm in the presence of CD4^+^CD25^+^ T cells/cpm in the absence of CD4^+^CD25^+^ T cells) × 100 %. The proliferation index (PI) was calculated using the formula: cpm after proliferation/cpm before proliferation.

### FoxP3 expression determined by real-time PCR

Foxp3 mRNA expression was quantified by real-time PCR using ABI PRISM 7700 Sequence Detector (Applied Biosystems). The human housekeeping gene β-Actin primers and probe set were used as the reference for sample normalization. Total RNA isolated from purified CD4^+^CD25^+^ T cell was reverse-transcribed into cDNA by using random hexamer primed. The primer set for Foxp3 was 5′-TTCGAAGAGCCAGAGGACTT-3′ and 5′-GCTGCTCCAGAGACTGTACC-3′. The probe for Foxp3 was 5′-FAM-CTCAAGCACTGCCAGGCGGACCATC-TAMRA-3′. The primer set for β-actin was 5′-ATCTGCTGGAAGGTGGACAGCGA-3′ and 5′-CCCAGCACAATGAAGATCAAGATCAT-3′. The probe for β-actin was 5′-FAM-TGAGCGCAAGTACTCCGTGTGGATCGGCG-TAMRA-3′(Invitrogen). The primers and probes used in the real-time PCR were ordered from Sangon (Shanghai, China) and designed not to amplify genomic DNA. Standard curves were generated from serial dilutions of purified plasmid DNA encoding the respective genes with a linear regression *R* greater than 0.99 and used to quantify mRNA copy numbers for each sample. The amplification protocol used was described as follows: 1 μl of synthesized cDNA product was subsequently added into PCR mix containing 25 μl of TaqMan 2 × PCR master mix (Applied Biosystems), 30 pmol human Foxp3 primer with 10 pmol probe, 2.5 μl β-Actin primer/probe set, and distilled water was added to make a total reaction volume of 50 μl. The PCR was programmed as an initial incubation for 10 min at 95 °C followed by 40 thermal cycles of 15 s at 95 °C and 1 min at 60 °C. The normalized values in each sample were calculated as the relative quantity of Foxp3 mRNA expression divided by the relative quantity of β-Actin mRNA expression. All reactions were confirmed by at least one additional independent run [18].

### Statistical analysis

Data were expressed as mean ± SD. CD4^+^CD25^−^ T cells, induced CD4^+^CD25^+^ T cells and nTregs were compared based on Bartlett’s test for homogeneity of variances. ANOVA and *t* test were used. *P* < 0.05 was considered as statistically significant difference.

## Results

### Frequencies of CD4^+^CD25^+^ Tregs in peripheral blood from MG patients

**Fig. 1:**
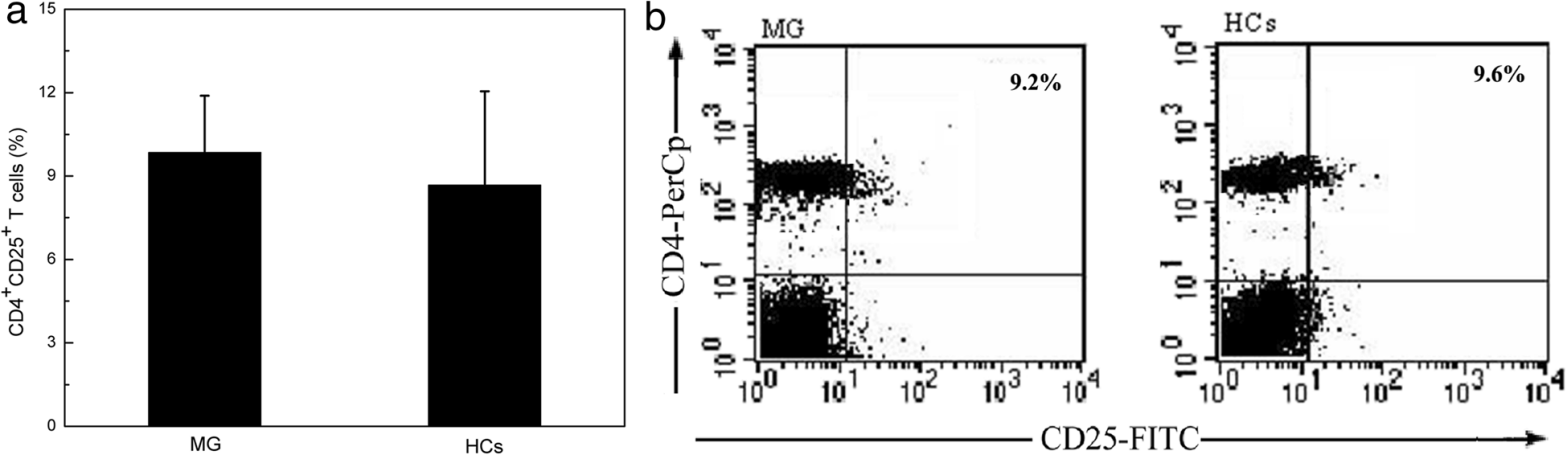
**a** Comparation of the percentages of CD4^+^CD25^+^ Tregs between MG patients and healthy controls (HCs). **b** Percentages of CD4^+^CD25^+^ T cells in MG patients and HCs. **a** MNCs are stained with PerCP-CD4 and PE-CD25. Gate was set on CD4^+^ T cell population. The data are from one experiment and are representative of 17 separate experiments. **b** The percentage of CD4^+^CD25^+^ T cells among CD4^+^ T cells in peripheral blood from MG patients is similar to that from HCs

In our experiment, Tregs were identified as CD4^+^CD25^high^ T cells by selecting those CD4^+^ cells whose CD25 expression exceeded the level of CD25 positivity observed on the CD4 negative population [19]. The number of CD4^+^CD25^+^ Tregs reservoir in peripheral blood was measured by flow cytometry. As shown in Fig. 1a, the percentages of CD4^+^CD25^+^ Tregs in peripheral blood from MG patients (9.85 ± 2.04 %) and HCs (8.67 ± 3.38 %) are similar (*P* > 0.05). Figure 1b is displayed as an example for a typical case study of one experiment representing the total 17 similar separate experiments.

### Effect of thymectomy on the number of CD4^+^CD25^+^ Tregs from MG patients

**Fig. 2:**
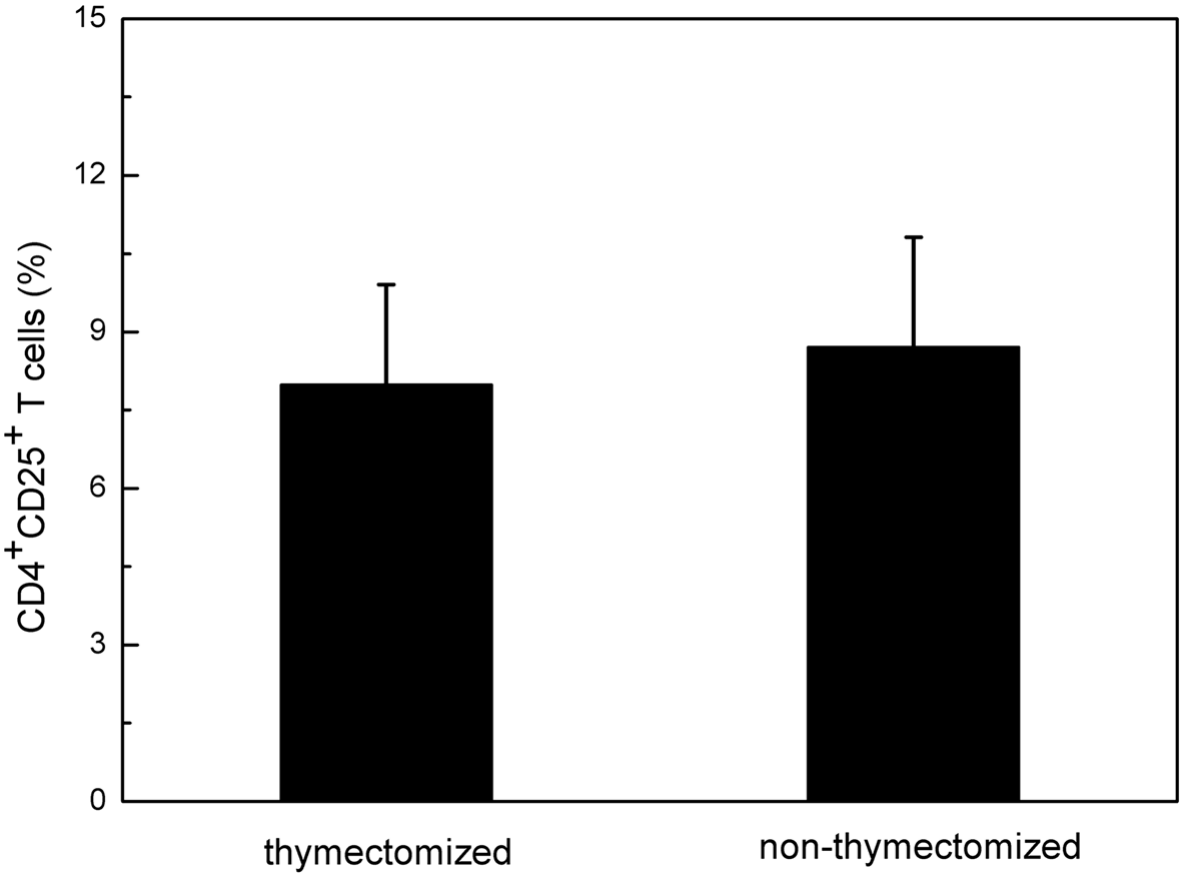
Comparation of the percentage of CD4^+^CD25^+^ Tregs between thymectomy and non- thymectomy MG patients

Twenty three MG patients were divided into two groups, including thymectomized and non-thymectomized MG patients. The influence of thymectomized on the number of CD4^+^CD25^+^ Tregs was investigated by comparing the percentages of CD4^+^CD25^+^ Tregs in CD4^+^ T cells between two groups. The results exhibit that the percentage of CD4^+^CD25^+^ Tregs is 7.99 ± 1.92 % in thymectomized group, while it is 8.71 ± 2.11 % in non-thymectomized group. It can be found that there is no statistically significant difference between them (*P* > 0.05) (Fig. 2).

### Function of CD4^+^CD25^+^ Tregs from MG patients

The proliferation of CD4^+^CD25^−^ T cells is expressed through the value of cpm and the PI is calculated by the formula: cpm after proliferation/cpm before proliferation. There is no significant difference between the proliferation of CD4^+^CD25^−^ T cells alone from MG patients (PI = 3.81 ± 0.63) and HCs (PI = 4.26 ± 0.77) (*P* > 0.05). These results suggest that the proliferation of CD4^+^CD25^−^ T cells with CD4^+^CD25^+^ Tregs from MG patients is higher than that of HCs. The PI from MG patients is 2.69 ± 0.63, while the PI from HCs is 1.52 ± 0.22. Such results indicate that CD4^+^CD25^+^ Tregs from MG patients are unable to effectively suppress the proliferation of their CD4^+^CD25^−^ T cells, so the suppressive function of CD4^+^CD25^+^ Tregs from MG patients is impaired.

### Conversion of induced CD4^+^CD25^+^ T cells from CD4^+^CD25^−^ T cells by IFN-γ

**Fig. 3:**
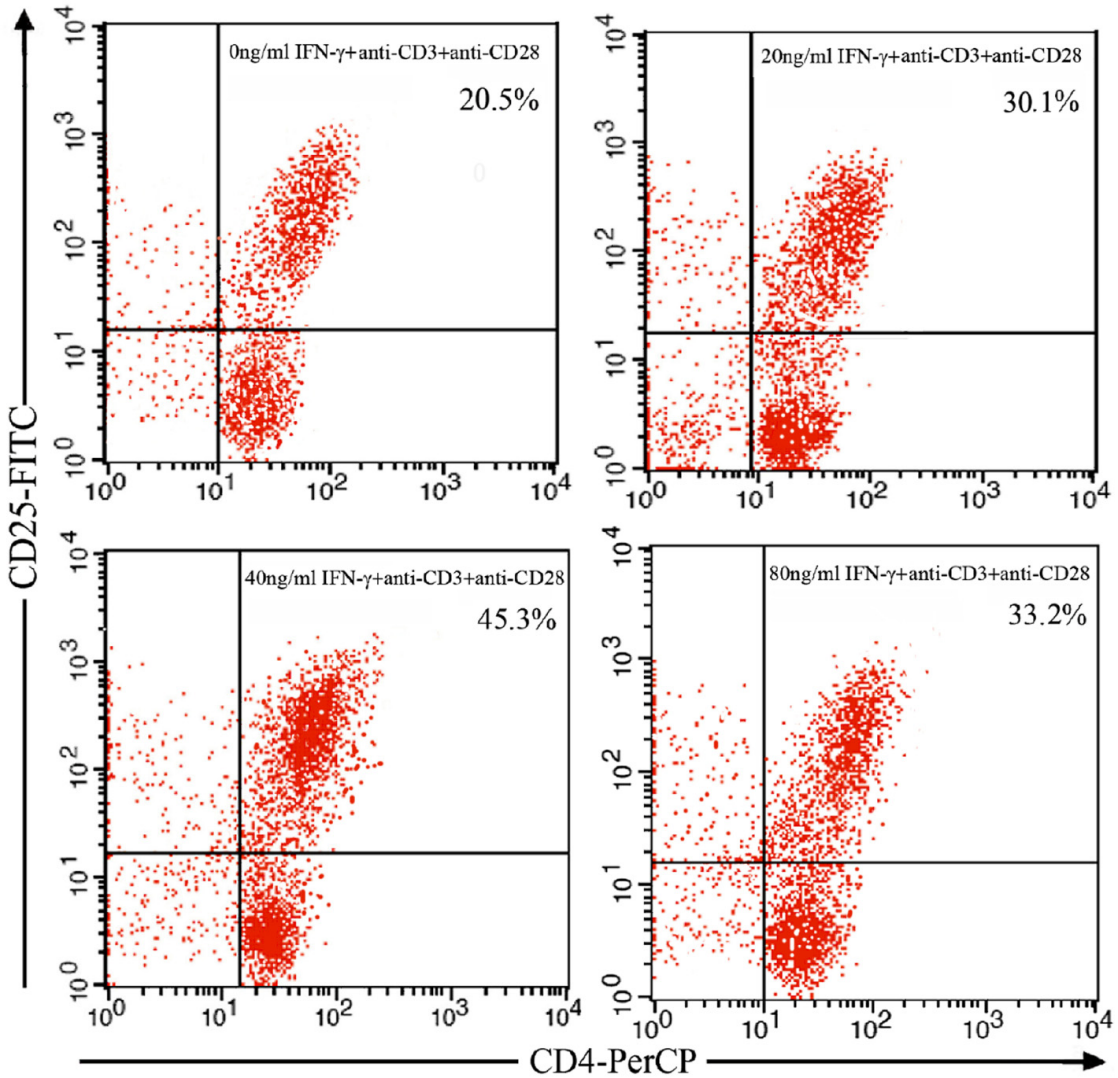
Percentages of the induced CD4^+^CD25^+^ T cells generated from CD4 + CD25ˉ T cells by IFN-γ with different concentrations in MG patients. CD4^+^CD25ˉT cells were stimulated by IFN-γ with different concentrations (0, 20, 40, 80 ng/ml) in the presence of anti-CD3 (1 μg/ml) and anti-CD28 (1 μg/ml) antibodies for 72 h and then sorted to measure the percentages of induced CD4^+^CD25^+^ T cells by flow cytometry. The data are from one experiment and are representative of 19 separate experiments

The conversion rate of the induced CD4^+^CD25^+^ T cells generated from CD4^+^CD25^−^ T cells changes with the concentration of IFN-γ in MG patients. The percentage of the induced CD4^+^CD25^+^ T cells firstly increases and later declines with increasing concentration of IFN-γ (*P* < 0.05). In the presence of anti-CD3 and anti-CD28 antibodies without IFN-γ, the induced CD4^+^CD25^+^ T cells can be generated from CD4^+^CD25^−^ T cells and the percentage of the induced CD4^+^CD25^+^ T cells is 18.3 ± 4.1 %. When the concentration of IFN-γ reaches as high as 40 ng/ml, the percentage of the induced CD4^+^CD25^+^ T cells has the highest value (43.1 ± 3.7 %). However, when the concentration of IFN-γ becomes higher as 80 ng/ml, the percentage of the induced CD4^+^CD25^+^ T cells decreases (29.8 ± 2.9 %) (Table 2). Figure 3 shows the percentages of the induced CD4^+^CD25^+^ T cells generated from CD4^+^CD25^−^ T cells by IFN-γ with different concentrations in MG patients. It is from one typical experiment result representing the total 19 separate experiments.

**Table 2:** Percentages of the induced CD4^+^CD25^+^ T cells by IFN-γ with different concentrations in MG patients (mean ± SD, *n* = 23)

IFN-γ (ng/ml)	0	20	40	80
CD4^+^CD25^+^ (%)	18.3 ± 4.1^**^	29.2 ± 3.1^*,**^	43.1 ± 3.7^*^	32.3 ± 4.5^*,**^

^*^ *P* < 0.05 vs. 0 ng/mL IFN-γ induced CD4^+^CD25^+^ T cells (%). ^**^ *P* < 0.05 vs. 40 ng/mL induced CD4^+^CD25^+^ T cells (%)

Our findings proved that the conversion of the induced CD4^+^CD25^+^ T cells from CD4^+^CD25^−^ T also occurred in HCs. When the concentration of IFN-γ is 20 ng/ml, the percentage of the induced CD4^+^CD25^+^ T cells is the highest in HCs. While the concentration of IFN-γ is 40 ng/ml, the percentage of the induced CD4^+^CD25^+^ T cells is nearly the same value. There is no statistical difference between them (Table 3).

**Table 3:** Percentages of the induced CD4^+^CD25^+^ T cells by IFN-γ with concentrations in healthy donors (mean ± SD, *n* = 19)

IFN-γ (ng/ml)	0	20	40	80
CD4^+^CD25^+^ (%)	32.8 ± 3.2^**^	55.2 ± 3.7^*^	54.1 ± 4.1^*^	42.8 ± 2.9^*,**^

*P < 0.05 vs. 0 ng/mL IFN-γ induced CD4^+^CD25^+^ (%). **P < 0.05 vs. 20 ng/mL IFN-γ induced CD4^+^CD25^+^ (%)

The results of MG patients and HCs are compared with every given concentration of IFN-γ separately by *t* test. It shows that with the same concentration of IFN-γ, the percentage of the induced CD4^+^CD25^+^ T cells from HCs varies from that of MG patients (*P* < 0.05). In particular, when the concentration of IFN-γ is 0 (only the anti-CD3 and anti-CD28 antibodies were added), the percentage of HCs also gives obviously higher value than that of MG patients. There is statistically considerable difference between them (*P* < 0.05).

The 23 MG patients details are shown in Table 1. No.1–7 are from those of 20–40 years old, No.8–14 are from those of 40–60 years old and No.15–23 are those over 60 years old. When the concentration of IFN-γ is 40 ng/ml, the percentages of the induced CD4^+^CD25^+^ T cells from the above three groups are 43.0 ± 3.5, 44.4 ± 3.6, 39.2 ± 4.1. And they are compared by ANOVA test. It has been found that the percentages have no statistical difference among them (*P* > 0.05).

### FoxP3 expression of induced CD4^+^CD25^+^ T cells

**Fig. 4:**
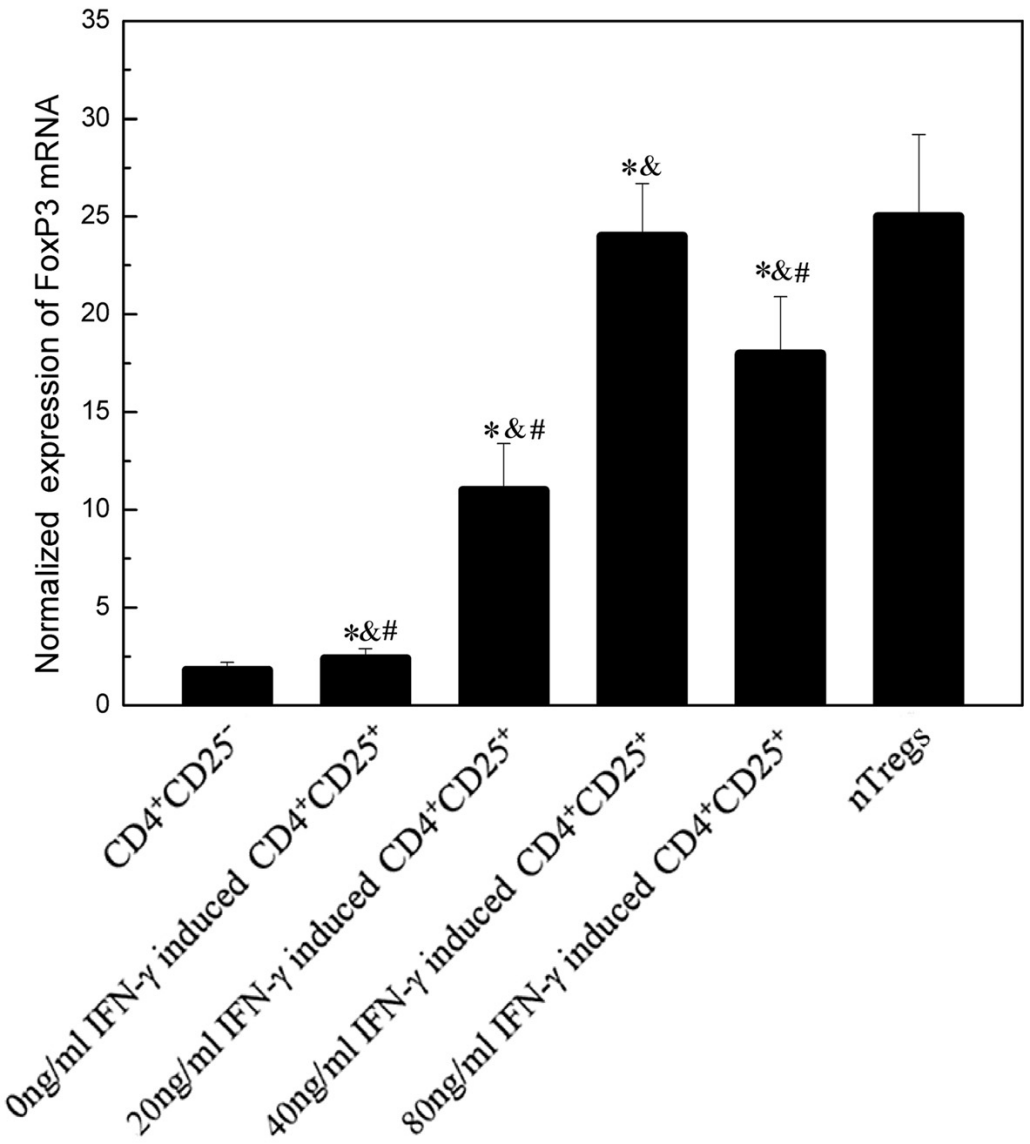
FoxP3 expression at mRNA levels in MG patients. FoxP3 mRNA expression of CD4^+^CD25ˉ T cells (before stimulating), the induced CD4^+^CD25^+^ T cells from MG patients and nTregs from HCs were assessed by real-time PCR (all the cells are purified). Results are expressed as mean ± SD, *n* = 15. ^*^, *P* < 0.05 vs. nTregs. &, *P* < 0.05 vs. CD4^+^CD25ˉ T cells (before stimulating) from MG patients. ^#^, *P* < 0.05 vs. 40 ng/mL induced CD4^+^CD25^+^ T cells

As described before, the CD4^+^ T cells which express the higher level of CD25 as the induced CD4^+^CD25^+^ T cells were selected [19]. But it should be noted that CD25 is not a specific marker of CD4^+^CD25^+^ Tregs. For example, Hong J and Leonard C found in that effector CD4^+^ T cells, CD25 can also be detected [20]. FoxP3 is the most specific marker in comparison with the other markers. Thus, besides CD25, FoxP3 is also included in our experiment detection to further study whether the induced CD4^+^CD25^+^ T cells are similar to nTregs phenotypically. Figure 4 shows the relationship between the FoxP3 expression of the induced CD4^+^CD25^+^ T cells at mRNA level and the concentration of IFN-γ in MG patients. It can be found that IFN-γ is able to induce FoxP3 expression. Even though the concentration of IFN-γ is lower (20 ng/ml), obvious induction of FoxP3 is observed. The FoxP3 expression reaches the highest when IFN-γ is 40 ng/ml (22 ± 3.1). But it is still lower than that in nTregs of HCs (25 ± 4.2) (*P* < 0.05).

### Suppressive function of induced CD4^+^CD25^+^ T cells

**Fig. 5:**
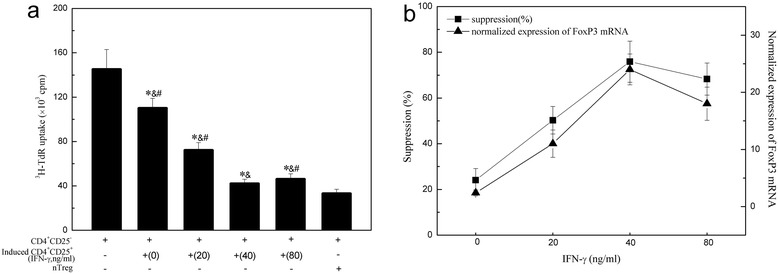
Suppressive function of the induced CD4^+^CD25^+^ T cells on the proliferation of autologous CD4^+^CD25ˉ T cells in MG patients. **a** The induced CD4^+^CD25^+^ T cells were co-cultured with autologous CD4^+^CD25ˉT cells at a 1:1 ratio to assay their suppressive function on the proliferation of CD4^+^CD25ˉ T cells. Results are expressed as mean cpm ± SD, *n* = 16. ^*^, *P* < 0.05 vs. CD4^+^CD25ˉ T cells cultured alone. &, *P* < 0.05 vs. co-cultures of CD4^+^CD25ˉT cells and nTregs. ^#^, *P* < 0.05 vs. 40 ng/mL induced CD4^+^CD25^+^ T cells. **b** IFN-γ dose-dependent induction of FoxP3 mRNA expression and the suppressive percentage of the induced CD4^+^CD25^+^T cells in suppressing proliferation of co-cultured autologous CD4^+^CD25ˉT cells. There is linear regression between them (r^2^ = 0.917, *p* = 0.029)

CD4^+^CD25^+^ Tregs have the ability to suppress autologous CD4^+^CD25^−^ T cells. So the suppressive function of the induced CD4^+^CD25^+^ T cells was evaluated to determine whether they are similar to nTregs functionally. In the presence of anti-CD3 antibody and irradiated APCs, the induced CD4^+^CD25^+^ T cells were co-cultured with autologous CD4^+^CD25^−^ T cells at a ratio of 1:1. In order to make a reasonable comparison, CD4^+^CD25^−^ T cells were cultured independently or co-cultured with nTregs (as the references). The suppressive function of the induced CD4^+^CD25^+^ T cells is estimated through the proliferative ability of autologous CD4^+^CD25^−^ T cells which is expressed as cpm (Fig. 5a). The value of cpm decreases when the induced CD4^+^CD25^+^ T cells are added to CD4^+^CD25^−^ T cells. The value of cpm is the highest when CD4^+^CD25^−^ T cells are cultured alone. The value is the lowest when the induced CD4^+^CD25^+^ T cells induced by 40 ng/ml IFN-γ are added and it is different from that of co-cultured CD4^+^CD25^−^ T cells and nTregs (33 ± 4.2) (*P* < 0.05). The value of cpm is equal to the proliferation of CD4^+^CD25^−^ T cells while this is opposite to the suppressive function of CD4^+^CD25^+^ Tregs. So this explains the CD4^+^CD25^+^ T cells induced by 40 ng/ml IFN-γ have the most powerful suppressive function but it is still lower than the function of nTregs.

The suppressive percentage was shown in Fig. 5b. It can be seen that the suppressive percentage is parallel to the relative FoxP3 mRNA expression as IFN-γ concentration increases. So FoxP3 mRNA expression plays an essential role in the development as well as the function of the induced CD4^+^CD25^+^ T cells.

## Discussion

MG might result from CD4^+^ T cell-dependent autoimmune response mediated by anti-AChR antibody, which is characterized by fluctuating weakness and easy fatigability of voluntary muscles. However, cellular and molecular mechanisms of MG remain unknown. Our recent studies show that CD4^+^CD25^+^ Tregs are critical in preventing autoimmune diseases by suppressing self-reactive T cells [2, 20, 21]. Since the pivotal role of CD4^+^CD25^+^ Tregs in maintaining the self-tolerance was indentified, our experiments were concentrated on whether the peripheral pool and the function of CD4^+^CD25^+^ Tregs are altered in MG patients. The majority of MG patients are also associated with the changes in the thymus, like hyperplasia or thymoma. Thus, thymic ablation has been used as a common therapy for many MG patients. Although thymectomy usually leads to symptomatic benefit or remission, the mechanism by which still needs further exploration. In the first part of the experiment, we found that there was no statistical difference for the number of CD4^+^CD25^+^ Tregs between MG patients and HCs. But the function of CD4^+^CD25^+^ Tregs from MG patients was defective compared with the function of nTregs. It is consistent with Balandina’s research [22]. Here we assimilated CD4^+^ T cells which express the higher level of CD25 as CD4^+^CD25^+^ Tregs the same as what Baecher-Allan did before [19]. It should be pointed out that we didn’t purify the CD4^+^CD25^+^ T cells to select the CD4^+^CD25^+^FoxP3^+^ T cells as Tregs. Many other Chinese studies show that the number of CD4^+^CD25^+^ Tregs in MG is decreased. The difference might be explained by that the CD4^+^CD25^+^ Tregs are defined in our study as CD4^+^CD25^high^ T cells while the other studies defined them as CD4^+^CD25^+^FoxP3^+^ Tregs [23]. In addition, after thymectomy, the number of CD4^+^CD25^+^ Tregs in MG patients had no statistical difference with the number of CD4^+^CD25^+^ Tregs in non-thymectomy MG patients. Our studies indicated that there may be other unknown parts or pathways to generate or differentiate CD4^+^CD25^+^ Tregs except for the thymus. So even after thymectomy, CD4^+^CD25^+^ Tregs can also be added to the peripheral blood to make up for a lack of CD4^+^CD25^+^ Tregs from the thymus.

Some experiments have shown that a large number of cytokines may be involved in generating CD4^+^CD25^+^ Tregs, such as TGF-β, IL-2 and et. al. Among the cytokines, IFN-γ is an important Th1 proinflammatory cytokine. However, some experiments show that IFN-γ may prevent mice from suffering autoimmune diseases. As a result, in this experiment, we focused our study on the relationship between IFN-γ and CD4^+^CD25^+^ Tregs in MG patients.

We found that IFN-γ with different concentrations can increase the percentage of CD4^+^CD25^+^ Tregs in the whole cell system. Because the purity of CD4^+^CD25^−^ T cells before stimulating is above 96 % and the viability of all the cells both before and after stimulating is above 95 %, we can confirm that the increased percentage of CD4^+^CD25^+^ Tregs is due to the conversion of CD4^+^CD25^−^ T cells by IFN-γ. Especially, in the presence of anti-CD3 and anti-CD28 antibodies only, CD4^+^CD25^+^ T cells can also be induced. It indicates anti-CD3 and anti-CD28 antibodies play an important role in helping IFN-γ to induce CD4^+^CD25^+^ T cells. Maybe anti-CD3 antibody provides a first signal through the TCR to activate CD4^+^CD25^−^ T cells and anti-CD28 antibody provides a costimulatory signal in this process. Moreover, induction ration is associated with the concentration of IFN-γ. When the concentration of IFN-γ is 40 ng/ml, the ration reaches the peak. The possible reason is that IFN-γ is a “double-edged sword”. It has regulatory and cytotoxic function. When the concentration of IFN-γ is between 20 and 40 ng/ml, it plays a regulatory role. Once it is above 40 ng/ml, it plays a cytotoxic role. In our study we show that 40 ng/ml IFN-γ has the most powerful ability to induce CD4^+^CD25^+^ Tregs in MG patients while the most suitable concentration of IFN-γ is 20 ng/ml in HCs. With IFN-γ of any concentration, the conversion rate of HCs is higher than that of MG patients. When anti-CD3 and anti-CD28 antibodies are added only, the rate of HCs is still higher than that of MG patients. It reveals that because the micro environments are different in MG patients and HCs, there may be some difference of sensitivity to IFN-γ in CD4^+^CD25^−^ T cells between MG patients and HCs or among the MG patients with different severity. And CD4^+^CD25^−^ T cells from HCs are also more sensitive to anti-CD3 and anti-CD28 antibodies. On the other side, the concentration of IFN-γ in plasma of MG may affect the function of CD4^+^CD25^+^ Tregs. The questions raised above will be studied in our subsequent experiments. The results from different age stages are also compared and these results show rarely any statistical difference. But there are other factors such as the severity of MG, sex and the duration of disease involved, so it is desirable to perform further study in the future to examine the exact effect of age to the induction.

The results provided above show that CD4^+^CD25^+^ T cells can be induced, but whether they have the similar phenotype as well as function requires further investigation. Since FoxP3 is the most specific marker for CD4^+^CD25^+^ Tregs, we choose it to identified whether the induced CD4^+^CD25^+^ T cells is CD4^+^CD25^+^ Tregs. FoxP3 can be expressed in the induced CD4^+^CD25^+^ T cells. Athough 40 ng/ml IFN-γ induced CD4^+^CD25^+^ T cells expressed the highest FoxP3 at mRNA level, it was still lower than the FoxP3 mRNA level of CD4^+^CD25^+^ T cells. We know the function of CD4^+^CD25^+^ Tregs relies on the FoxP3 expression. Because some activated CD4^+^ T cells express CD25, maybe it is more reasonable that the CD4^+^CD25^+^ Tregs are identify as CD4^+^CD25^+^FoxP3^+^ T cells. Then, we analyzed the suppressive function of the induced CD4^+^CD25^+^ T cells. The 40 ng/ml IFN-γ induced CD4^+^CD25^+^ T cells most significantly suppress the proliferation of CD4^+^CD25^−^ T cells, whereas it is still defective than the suppressive function of nTregs. The phenotypical and functional difference between nTregs and the induced CD4^+^CD25^+^ T cells may be due to the activated CD4^+^ T cells which express CD25 involved in the induced CD4^+^CD25^+^ T cells. So the induced CD4^+^CD25^+^FoxP3^+^ T cells are the actual parts that are similar to CD4^+^CD25^+^ Tregs. The calculated suppressive ration is associated with the FoxP3 mRNA expression. It reveals that the function of the induced CD4^+^CD25^+^ T cells depends on the expression of FoxP3. It provides evidence that the suppressive function of CD4^+^CD25^+^ Tregs may be mainly attributed to FoxP3-positive CD4^+^CD25^+^ Tregs [24]. It is consistent with the first part of our experiment

## Conclusions

MG patients have the similar number of CD4^+^CD25^+^ T cells to healthy controls but the function of CD4^+^CD25^+^ T cells from MG patients is defective. It is likely some CD4^+^CD25^+^ T cells are FoxP3 negative which has no suppressive function. The number of CD4^+^CD25^+^ T cells has no difference between the thymectomy and no-thymectomy MG patients. IFN-γ can induce CD4^+^CD25^−^ T cells from MG patients to CD4^+^CD25^+^ T cells in vitro and 40 ng/ml is the most appropriate concentration.

Our study provides the definitive evidence for IFN-γ inducing CD4^+^CD25^−^ T cells to CD4^+^CD25^+^ Tregs in MG patients in vitro. This subject will further reveal the role of IFN-γ in the pathogenesis of MG from a new perspective. It will also provide the scientific basis for the clinical targeted therapy of MG.
